# Fabrication of Metal and Metal Oxide Nanoparticles by Algae and their Toxic Effects

**DOI:** 10.1186/s11671-016-1580-9

**Published:** 2016-08-17

**Authors:** Khwaja Salahuddin Siddiqi, Azamal Husen

**Affiliations:** 1 Department of Chemistry, Aligarh Muslim University, Aligarh, 202002 Uttar Pradesh India; 2 Department of Biology, College of Natural and Computational Sciences, University of Gondar, P.O. Box #196, Gondar, Ethiopia

**Keywords:** Metal and Metal Oxide Nanoparticles, Controlling Factors, Biosynthesis, Characterization and Mechanism, Antimicrobial Activity

## Abstract

Of all the aquatic organisms, algae are a good source of biomolecules. Since algae contain pigments, proteins, carbohydrates, fats, nucleic acids and secondary metabolites such as alkaloids, some aromatic compounds, macrolides, peptides and terpenes, they act as reducing agents to produce nanoparticles from metal salts without producing any toxic by-product. Once the algal biomolecules are identified, the nanoparticles of desired shape or size may be fabricated. The metal and metal oxide nanoparticles thus synthesized have been investigated for their antimicrobial activity against several gram-positive and gram-negative bacterial strains and fungi. Their dimension is controlled by temperature, incubation time, pH and concentration of the solution. In this review, we have attempted to update the procedure of nanoparticle synthesis from algae, their characterization by UV-vis, Fourier transform infrared spectroscopy, transmission electron microscopy, scanning electron microscopy, x-ray diffraction, energy-dispersive x-ray spectroscopy, dynamic light scattering and application in cutting-edge areas.

## Review

### Introduction

The nanoparticles are the most fundamental component in the fabrication of a nanostructure. Several synthetic routes are used for the fabrication of nanoparticles of diverse morphology and size. Although these procedures have offered superior quality of nanoparticles, better fabrication procedures are yet to be developed. Currently, scientists have focused their attention on the biosynthesis of nanoparticles involving plant, algae, bacteria, fungi and virus containing proteins, amines, aminoacids, phenols, sugars, ketones and aldehydes which act as reducing agents, capping agents and stabilizers for nanoparticles [1–8].

**Fig. 1:**
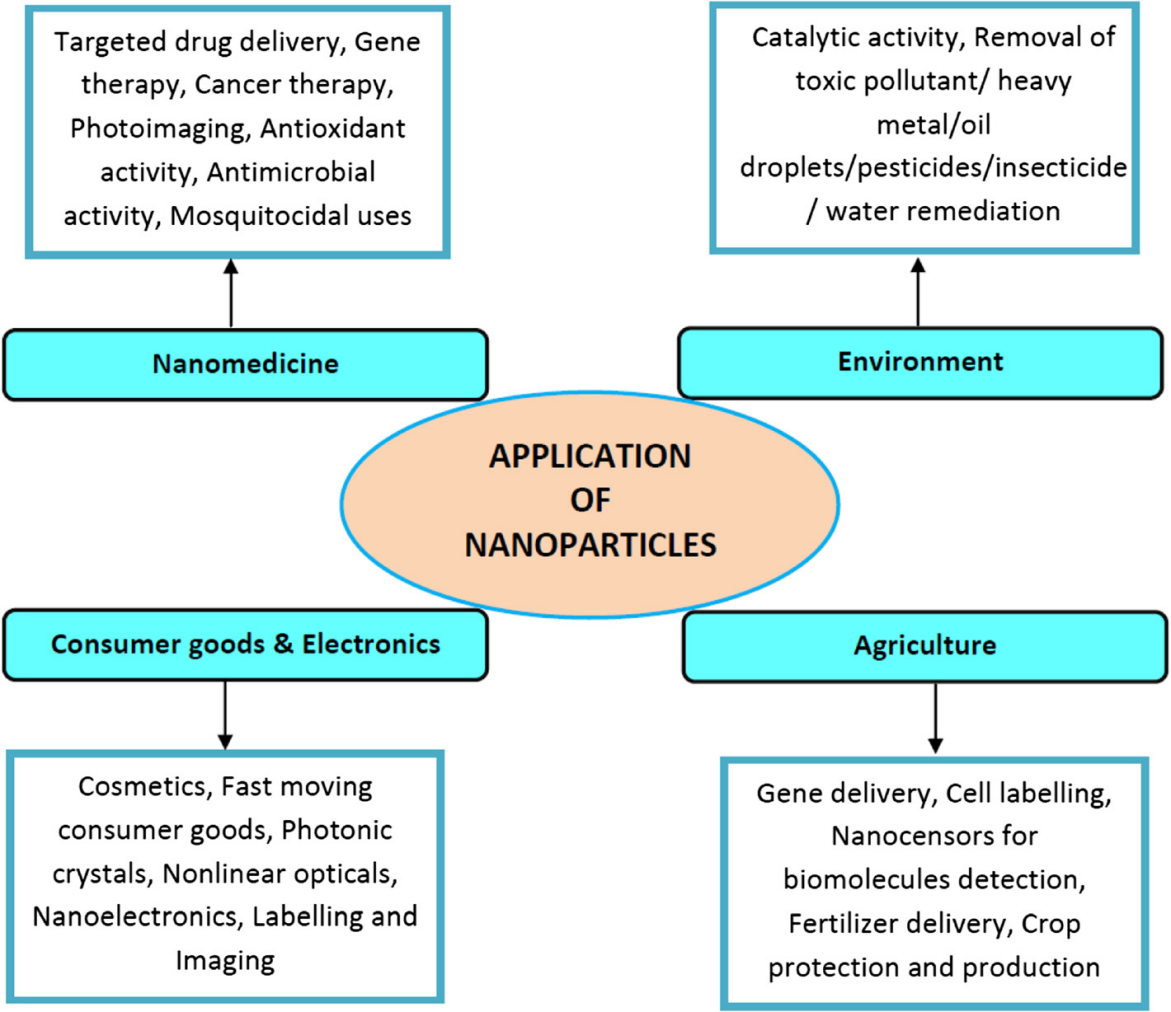
Application of fabricated nanoparticles in cutting-edge areas

The use of algae for biogenic synthesis of nanoparticles has become prevalent during these days due to their easy access and efficacy [9–11]. The biomolecules present in the algal extract have relatively been less exploited for nanoparticle synthesis than similar other natural sources such as plants and bacteria [12, 13]. Available functional groups and enzymes in the algal cell walls act as reducing agents, as a consequence of which reduction and fabrication of metal and metal oxide nanoparticles occur at ambient conditions [14, 15]. In recent days, several diverse and potential applications of nanoparticles in crop protection and production, cosmetics, drug delivery, photonic crystals, analysis, food, coatings, paints, bioremediation, catalysis and material science have been applied [4, 7, 16, 17] (Fig. 1). However, the mechanism of interaction of nanoparticles with biological systems at the molecular level is not clearly understood [17–19]. It is essential to understand the intricacies of the various steps involved in the fabrication of nanoparticles from algae, their antimicrobial activity and impact on the environment. Among the transition metal nanoparticles, gold and silver have received more attention than others owing to their application in drug delivery, tumor imaging, identification of pathogens and determination of heavy metals [20–22].

Algae are a rich source of biomolecules and frequently used for the extracellular synthesis of nanoparticles [10, 23–25].

Metal nanoparticles have gained extensive attention due to their efficient antimicrobial activities because they can be safely used in human system to inhibit the growth of pathogens without damaging the normal tissues. The mechanism of antimicrobial activity of nanoparticles has been ascribed to the generation of free radical and subsequent damage of the microbial cell wall leading to their death [26]. Besides, the nanoparticles poison the enzyme of single cell pathogens such as bacteria, fungi and viruses for oxygen intake without harming the human enzymes [27]. Algae may produce nanoparticles from any metal salt by extracellular or intracellular pathways involving biochemicals or enzymes present in them. However, enzymes and reducing substances are known to be the main constituents of microorganisms and fungi for the production of metal nanoparticles from metal salts [7, 28–30].

**Fig. 2:**
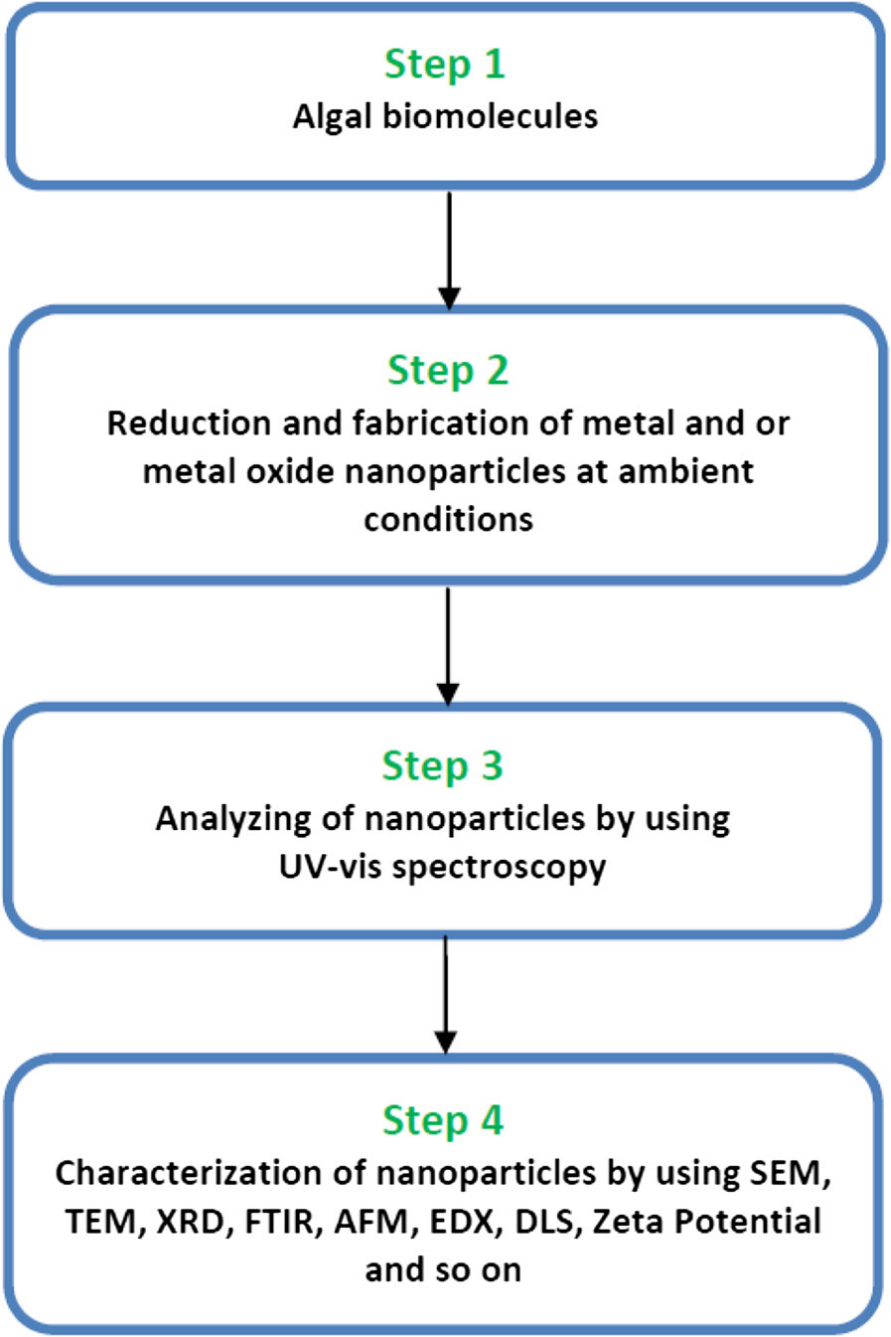
Fabrication/synthesis of nanoparticles from algal molecules and its characterization

Synthesis of metal and metal oxide nanoparticles of well-defined shape and size depends on the concentration of algal extract/biomass, metal salt, pH of the reaction mixture, temperature and incubation time. They can be characterized by UV-vis, Fourier transform infrared (FTIR), transmission electron microscopy (TEM), scanning electron microscopy (SEM), x-ray diffraction (XRD), energy-dispersive x-ray spectroscopy (EDX) and dynamic light scattering (DLS) (Fig. 2). Biogenic fabrication of metal and metal oxide nanoparticles using various algal species such as *Bifurcaria bifurcate*, *Chlamydomonas reinhardtii*, *Chlorella vulgaris*, *Ecklonia cava*, *Fucus vesiculosus*, *Oscillatoria willei*, *Pithophora oedogonia*, *Sargassum muticum*, *Sargassum wightii*, *Spirulina platensis*, *Stoechospermum marginatum* etc. are presented in Table 1. Both freshwater and marine algae have given impetus to the development of industry and technology alike as they prevent pollution in the atmosphere. However, it is quite obvious that nanoparticles may have a positive or negative impact in the living system depending on their shape, size and above all the nature of specific metal ion.

**Table 1:** Engineered nanoparticles of varying size and shape fabricated from various algal species

Nanoparticles	Algal Species	Size (nm)	Shape	References
Gold	*Sargassum wightii*	8–12	–	Singaravelu et al. [10]
	*Sargassum muticum*	5.42 ± 1.18	Spherical	Namvar et al. [31]
	*Spirulina platensis*	6–10	–	Govindaraju et al. [32]
	*Spirulina platensis*	~5	–	Uma Suganya et al. [33]
	*Stoechospermum marginatum*	18.7–93.7	Spherical and Hexagonal	Rajathi et al. [34]
	*Navicula atomus*	9	–	Schröfel et al. [35]
	*Cladosiphon okamuranus*	8.54–10.74	–	Lirdprapamongkol et al. [36]
	*Tetraselmis kochinensis*	5–35	Spherical and Triangular	Senapati et al. [37]
	*Ecklonia cava*	30 ± 0.25	Spherical and Triangular	Venkatesan et al. [38]
	*Chlorella vulgaris*	2–10	Spatial array of Self Assembled Structures	Annamalai and Nallamuthu [39]
	*Padina gymnospora*	53–67	Spherical	Singh et al. [40]
	*Fucus vesiculosus*	Varied	Spherical	Mata et al. [41]
	*Turbinaria conoides*	2–19	Triangular	Vijayan et al. [42]
Silver	*Spirulina platensis*	7–16	–	Govindaraju et al. [32]
	*Oscillatoria willei*	100–200	–	Mubarak Ali et al. [43]
	*Caulerpa racemosa*	5–25	Spherical and Triangular	Kathiraven et al. [44]
	*Cystophora moniliformis*	50–100	Spherical	Prasad et al. [45]
	*Chlamydomonas reinhardtii*	5–35	Round and Rectangular	Barwal et al. [46]
	*Turbinaria conoides*	2–17	Spherical	Vijayan et al. [42]
	*Pithophora oedogonia*	25–44	Cubical and Hexagonal	Sinha et al. [47]
	*Caulerpa racemosa*	5–25	–	Kathiraven et al. [44]
Copper Oxide	*Bifurcaria bifurcata*	5–45	Spherical	Abboud et al. [48]
Zinc Oxide	*Sargassum muticum*	30–57	Hexagonal	Azizi et al. [49]
Iron Oxide	*Sargassum muticum*	18 ± 4	Cubic	Mahdavi et al. [50]

In this review article, we have discussed the recent advances in nanoparticle fabrication techniques from algae, their characterization by UV-vis, FTIR spectroscopy, TEM, SEM, XRD, AFM, EDX, DLS and application as antimicrobial agents.

### Metal Nanoparticles

**Fig. 3:**
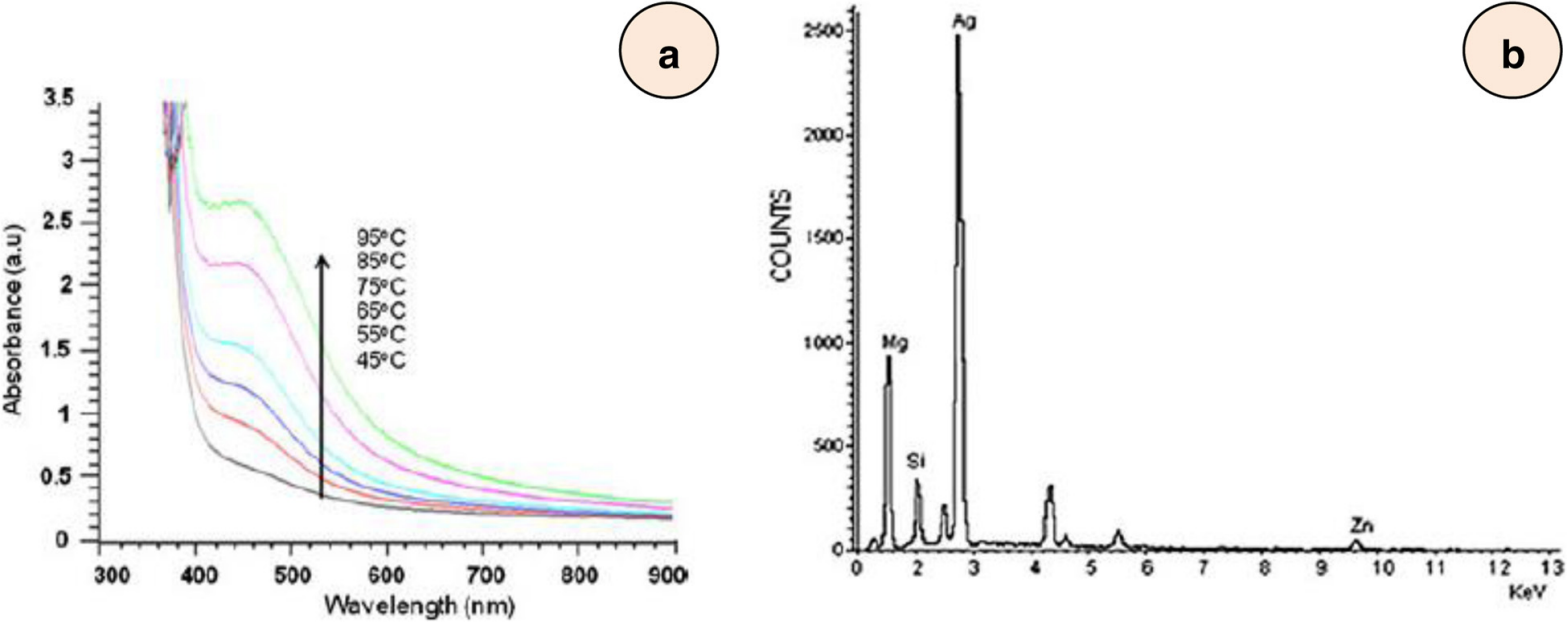
**a** UV absorption spectra and (**b**) EDAX analysis of silver nanoparticles synthesized using *Cystophora moniliformis* [45]

Silver nanoparticles have been synthesized from *Cystophora moniliformis* algal extract in aqueous medium at 65 °C [45]. It has been noted that with an increase in temperature, the size of the nanoparticles increases which may be confirmed from their UV-vis spectra (Fig. 3a). The surface plasmon resonance (SPR) peak slowly sharpens with temperature and becomes stable between 65 and 75 °C. It has been suggested that the peak between 450 and 452 nm corresponds to polydispersed spherical silver nanoparticles. Although the SPR peak shifts towards longer wavelength with increasing temperature, the formation of nanoparticles becomes faster but their aggregation occurs between 85 and 95 °C. The size of silver nanoparticles varies between 50 and 100 nm (Fig. 3b).

Biosynthesis of silver nanoparticles from polysaccharides extracted from four marine algae namely, *Pterocladia capillacae*, *Jania rubins*, *Ulva faciata* and *Colpmenia sinusa* has been reported [1]. They were found to be spherical with 7–20 nm diameter. Their antibacterial activity has been ascribed to their attachment to bacterial cell wall inhibiting their vital functions.

Fabrication of silver nanoparticles from *Sargassum longifolium* alga and their microbial activity against several pathogens have been reported [51]. The yellow reaction mixture comprising of AgNO_3_ and aqueous algal extract turned brown after 1 h. However, the reaction was completed after 32 h, the intensity of which is time dependent. The absorption peak at 440 nm indicated the formation of polydispersed silver nanoparticles. It has been reported that the pH of the reaction mixture exhibited a significant role in the silver nanoparticle synthesis. The colour change of the reaction mixture was slower at low pH 6.2 than that at high pH 8.4. The colour intensity of the reduction process was increased with the increase of the pH. The antifungal activity against *Aspergillus fumigatus*, *Candida albicans* and *Fusarium* sp. was found to increase with increasing concentration of silver nanoparticles [51].

Biosynthesis and antibacterial activity of silver nanoparticles of 25–44 nm diameter using fresh water green alga, *Pithophora oedogonia*, has been reported. IR spectrum and quantitative analysis of the extract showed the presence of carbohydrates, saponins, steroids and proteins which reduce AgNO_3_ to silver nanoparticles. They were found to be more effective against gram negative bacteria than gram positive ones [47].

Kathiraven et al. [44] have also reported the biosynthesis of silver nanoparticles from marine alga, *Caulerpa racemosa* and their antibacterial activity against human pathogens. They were (silver nanoparticles of 5–25 nm) crystalline with face-centred cubic geometry and effective against *Staphylococcus aureus* and *Proteus mirabilis* bacteria at a very low concentration (5–15 μL). Silver nanoparticles were synthesized from 14 bacteria and microalgae. It was observed that the nanoparticles were produced by extracellular polysaccharides even in the dark. Spherical, elongated and irregular silver nanoparticles of different dimensions and morphology were obtained which vary from one species to another [5]. The antibacterial activity was tested against six pathogenic bacteria. The mechanism involves free radical formation which causes damage to the cellular membrane.

Small gold nanoparticles of uniform shape with an average size of ~5 nm were obtained from blue green alga, *Spirulina platensis* [33]. The protein extract of alga and HAuCl_4_ in a 1:1 ratio in the presence of NaOH was incubated at room temperature for 48 h. Colour change from green to greyish yellow and eventually to ruby red showed the formation of gold nanoparticles [52]. Three distinct peaks at 685, 524 and 385 nm were observed along with an excitation maximum at 620 nm. The peaks at 685 and 629 nm assigned to HOMO and LUMO charge transfer transitions [53] are the frequencies for secondary amines, OH and COO^−^ groups which would have stabilized the gold nanoparticles. Their antibacterial activity against *Bacillus subtilis* was examined. The results indicated that nanoparticles caused damage to cells by producing pits in the outer cell wall which disrupt the normal functioning of the bacteria [34]. Since the gold nanoparticles are smaller than the thickness of bacterial cell wall they can easily penetrate into the cell and inhibit their growth.

Extracellular biosynthesis of gold nanoparticles using marine alga *Sargassum wightii* of 8–12 nm has been reported [10]. The reaction was completed in 15 h with a visibly distinct ruby colour with an absorption maximum at 527 nm. The TEM images showed monodispersed gold nanoparticles, where they are predominantly sphere of 11 nm.

Parial et al. [54] have reported the fabrication of gold nanoparticles from three cynobacteria (*Phormidium valderianum*, *Phormidium tenue*, *Microcoleus chthonoplastes*) and four green algae (*Rhizoclonium fontinale*, *Ulva intestinalis*, *Chara zeylanica*, *Pithophora oedogoniana*) at different pH at 20 °C. Generally, the gold nanoparticles were spherical at neutral pH and at pH 9 along with hexagonal and triangular ones. At pH 7 and 9, they exhibited a single absorption between 520 and 534 nm, while at about pH 5, two absorption bands at 520 and one ~600–670 were observed. The peaks vary with pH, concentration of the solution and the nature of cynobacteria and algae. These factors also affect the shape and size of the gold nanoparticles. At pH 5, the small spherical particles (15 nm) together with nano rods (411 × 32 nm) with some larger ones (17 nm) are produced. It is, however, noted that all gold nanoparticles are monodispersed with some aggregation.

Dahoumane and co-workers [8] have synthesized gold nanoparticles from living cells of *Euglena gracilis* microalga. The biomaterial in the alga act as reducing agent, capping agent and catalyst similar to other marine algae [55]. The pH, reaction time, temperature and concentration are controlling factors for the nanoparticles yield. It has been proposed that gold nanoparticle formation and release occur in three steps: (1) uptake of Au^+3^, (2) reduction of Au^+3^ to Au^0^ and (3) release of gold nanoparticles into the solvent. They are well dispersed and do not aggregate. They are spherical in shape whose dimensions vary from 10 nm to several 100 nm. AuCl_3_ concentration of 10^−3^ M is lethal to *E. gracilis* which suggests that all algae have a tolerance limit and certain capacity to reduce metal ions to protect themselves from the toxic influence of Au^3+^/Au^0^.

Biogenic fabrication of gold nanoparticles by brown alga, *Stoechospermum marginatum* biomass, has been reported [34]. The brown colour of extract turned ruby red within 10 min of addition of HAuCl_4_ exhibiting an absorption at 550 nm in UV-vis spectrum due to SPR [10]. The TEM image revealed that majority of the polydispersed nanoparticles were spherical, hexagonal and triangular with size ranging between 18.7 and 93.7 nm. However, SEM images showed the formation of gold nanoparticles of 40–85 nm. Since the algal extract is known to contain terpenoids and phenols, they reduce the gold ions to gold nanoparticles which are reflected from a change in colour. X-ray diffraction pattern showed face-centred cubic gold structure [56]. Their antimicrobial activity was nearly half of the tetracycline (Table 2) but it is higher than tetracycline against *Enterobacter faecalis*.

**Table 2:** Antibacterial activity of gold nanoparticles (modified, [34])

Bacterial Pathogens	Gold Nanoparticles	Positive Control (tetracycline)	Negative Control (chloroauric acid)
*Pseudomonas aeruginosa*	8	13	0
*Klebsiella oxytoca*	7	14	0
*Enterobacter faecalis*	11	9	0
*Klebsiella pneumoniae*	6	12	0
*Vibrio cholerae*	8	15	0
*Escherichia coli*	0	12	0
*Salmonella typhii*	6	13	0
*Salmonella paratyphi*	8	13	0
*Vibrio parahaemolyticus*	9	17	0
*Proteus vulgaris*	8	14	0

**Fig. 4:**
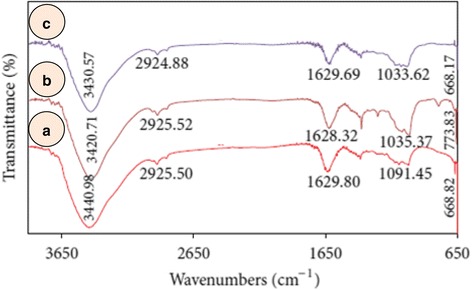
FTIR spectra of (**a**) *Turbinaria conoides* extract (**b**) silver and (**c**) gold nanoparticles [42]

Vijayan et al. [42] have reported the fabrication of gold and silver nanoparticles from a seaweed called *Turbinaria conoides*. They have been thoroughly characterized, and their antimicrofouling activity has also been evaluated. There are certain microbes which attach themselves to a solid support by producing extracellular polymeric materials in the form of a thin biofilm to which many other fouling agents are attached. In the case of ships, such thin films progressively become thick, increase the weight of the ship, corrode the metal and produce a foul smell. FTIR spectra (Fig. 4) showed peaks corresponding to OH, C=O and C-OH functional groups, but the exact compound containing these groups have not been identified. However, alcohol or ketone may act as a reducing agent but the authors have wrongly taken OH as a hydroxyl group and later identified as an alcoholic group. Likewise, they took the ketonic group C=O as a carboxylic group and suggested them as reductant. Their assignment of the functional groups is based on wrong assumption and is therefore highly dubious. Silver nanoparticles were found to be effective in controlling the bacterial biofilm formation, whereas gold nanoparticles were completely ineffective. Since silver nanoparticles are toxic to many microbes, they can be used to inhibit their growth in vitro and in vivo irrespective of their size, but nontarget organisms may also be affected.

**Fig. 5:**
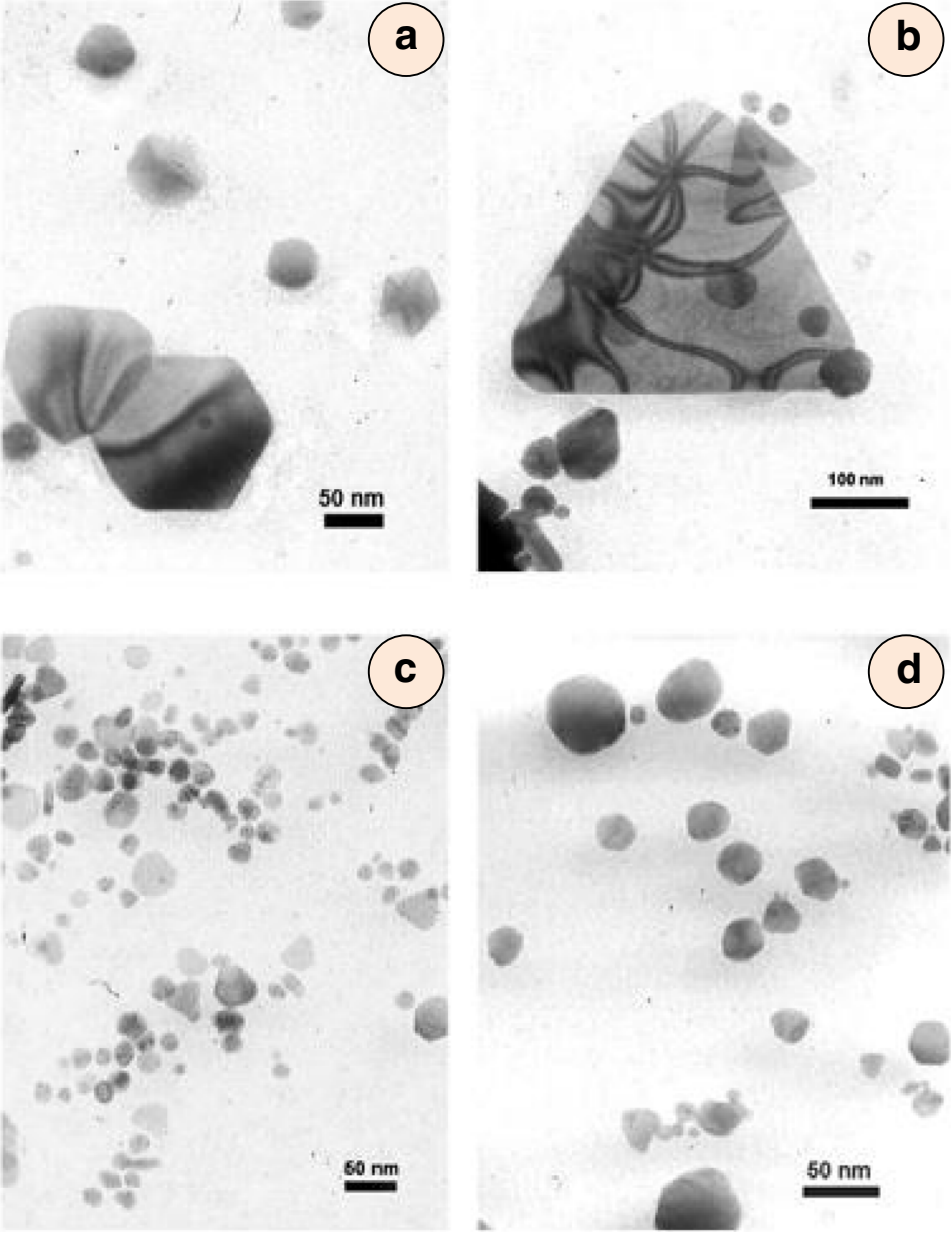
TEM images of gold nanostructures synthesized using *Chondrus crispus* at different initial pH values. **a** Detail of hexagonal nanoparticles obtained at pH 2. **b** Detail of a nanotriangle obtained at pH 2, **c** pH 4 and **d** pH 10 [57]

Nanobiotechnology is extremely useful in exploiting potential of algae and microbes to convert small quantity of metal from huge deposits of ores. Gold and silver nanoparticles were synthesized from red *Chondrus crispus* and *Spyrogira insignis* algae [57]. The structure and size were found to be dependent on pH of the solution between 2 and 10. The yield of gold nanoparticles was 70 % at pH 2 but it decreased with increasing pH and, at pH 10, the yield was nearly 60 % only. TEM images revealed that gold nanoparticles produced in acidic medium were polygonal, triangular and hexagonal (Fig. 5). An increase in pH from 2 to 4 showed decrease in size of gold nanoparticles (~30 nm). Formation of spherical nanoparticles was detected from a change in UV-vis absorption spectra which correspond to different shapes. Thus, polygonal nanoparticles or nanosphere may be produced simply by changing the pH of the reaction mixture. However, the UV-vis spectra slightly change due to the colour of the algae too. Kuyucak and Volesk [58] have suggested the following reaction to occur for the reduction of gold ions to gold nanoparticles.

$$ {\mathrm{Au}\mathrm{Cl}}_{4^{-}}\kern0.5em +\kern0.5em 3\mathrm{R}\kern0.5em -\kern0.5em \mathrm{O}\mathrm{H}\kern1.5em \to \kern1.5em {\mathrm{Au}}^{\mathrm{o}}\kern0.5em +\kern0.5em 3\mathrm{R}\kern0.5em =\kern0.5em \mathrm{O}\kern0.5em +\kern0.5em 3{\mathrm{H}}^{+}\kern0.5em +\kern0.5em 4{\mathrm{Cl}}^{-} $$

This equation is not balanced because it does not account for 3H^+^ with 4Cl^−^. It should be written as follows:

$$ {\mathrm{Au}\mathrm{Cl}}_{4^{-}}\kern0.5em +\kern0.5em 4\mathrm{R}\kern0.5em \hbox{-} \hbox{-} \kern0.5em \mathrm{O}\mathrm{H}\kern1.5em \to \kern1.5em {\mathrm{Au}}^{\mathrm{o}}\kern0.5em +\kern0.5em 4\mathrm{R}\kern0.5em =\kern0.5em \mathrm{O}\kern0.5em +\kern0.5em 4{\mathrm{H}}^{+}\kern0.5em +\kern0.5em 4{\mathrm{Cl}}^{-}. $$

Alternatively, it can be written in the following form:

$$ {\mathrm{H}\mathrm{AuCl}}_{4^{-}}\kern0.5em +\kern0.5em 3\mathrm{R}\mathrm{O}\mathrm{H}\kern1.5em \to \kern1.5em {\mathrm{Au}}^{\mathrm{o}}\kern0.5em +\kern0.5em 3\mathrm{R}\kern0.5em =\kern0.5em \mathrm{O}\kern0.5em +\kern0.5em 4{\mathrm{H}}^{+}\kern0.5em +\kern0.5em 4{\mathrm{Cl}}^{-}. $$

### Metal Oxide Nanoparticles

Biosynthesis of zinc oxide nanoparticle from aqueous extract of brown marine macroalga, *Sargassum muticum* has been reported [49]. The colour of the reaction mixture containing ZnO and algal extract changed from dark brown to a pale white colour indicating the synthesis of zinc oxide nanoparticle. Surface and hydroxyl moieties of polysaccharide present in the extract are involved in the formation of zinc oxide nanoparticles of 30–57 nm. They were agglomerated with hexagonal structure. Authors have concluded that the synthesized zinc oxide nanoparticles prepared from *S. muticum* is expected to have notable applications in pharmaceutical and biomedical fields and in cosmetic industries.

Abboud et al. [48] have reported the synthesis of copper oxide nanoparticles of 5–45 nm dimension from *B. bifurcate* algal extract. They were shown to be a mixture of Cu(I) and Cu(II) oxides and were crystalline in nature. Transition metal oxide nanoparticles are an important class of semiconductors and because of incompletely filled d orbitals, they find application in magnetic storage media, energy transformation, electronic and catalysis [59–61]. The formation of copper oxide nanoparticles was confirmed by a change in colour when 1 mM solution of CuSO_4_ was added to *B. bifurcate* extract at ambient temperature. Their UV-vis spectra showed distinct change in the absorption peaks owing to the presence of diterpenoids in the extract followed by the formation of cuprous oxide and cupric oxide nanoparticles [62, 63]. The CuSO_4_ undergoes partial reduction to Cu(I) and Cu(II) oxides which is reflected from the blood red colour exhibiting absorption at 260 and 650 nm. The TEM image showed that majority of the nanoparticles are spherical, although some elongated ones were also observed. Since the nanoparticles are a mixture of cupric oxide and cuprous oxide the XRD pattern showed the presence of two crystalline phases, monoclinic copper(I) oxide and copper(II) oxide with cuprite structure. The antibacterial activity of algal extract and copper oxide nanoparticles was tested against *Enterobacter aerogenes* and *Staphylococcus aureus*. It was observed that the algal extract alone was ineffective while copper oxide nanoparticles were significantly active against two bacterial strains.

Iron oxide nanoparticles were synthesized from FeCl_3_ with an aqueous extract of brown alga *Sargassum muticum* at 25 °C. The polysaccharides present in the algal extract reduce the FeCl_3_ to Fe_3_O_4_ nanoparticles of 18 ± 4 nm size which are mainly cubic in shape [50].

Very few reports are available on the toxic effects of several metal nanoparticles on marine organisms including algae, bacteria and protozoa in order to have a data bank for risk assessment [64]. Aruoja et al. [65] have synthesized Al_2_O_3_, Co_3_O_4_, CuO, Fe_3_O_4_, MgO, Mn_3_O_4_, Sb_2_O_3_, SiO_2_, ZnO, TiO_2_, WO_3_ and Pd crystalline nanoparticles. They are 8–21 nm in size. Some of these oxide nanoparticles are acidic, some are basic and others are amphoteric in nature. They give stable suspension in water. Their toxicity has also been investigated against one alga (*Pseudokirchneriella subcapitata)*, three bacteria (*Vibrio fischeri*, *Escherichia coli*, *Staphylococcus aureus*) and one protozoa (*Tetrahymena thermophila)*.

**Fig. 6:**
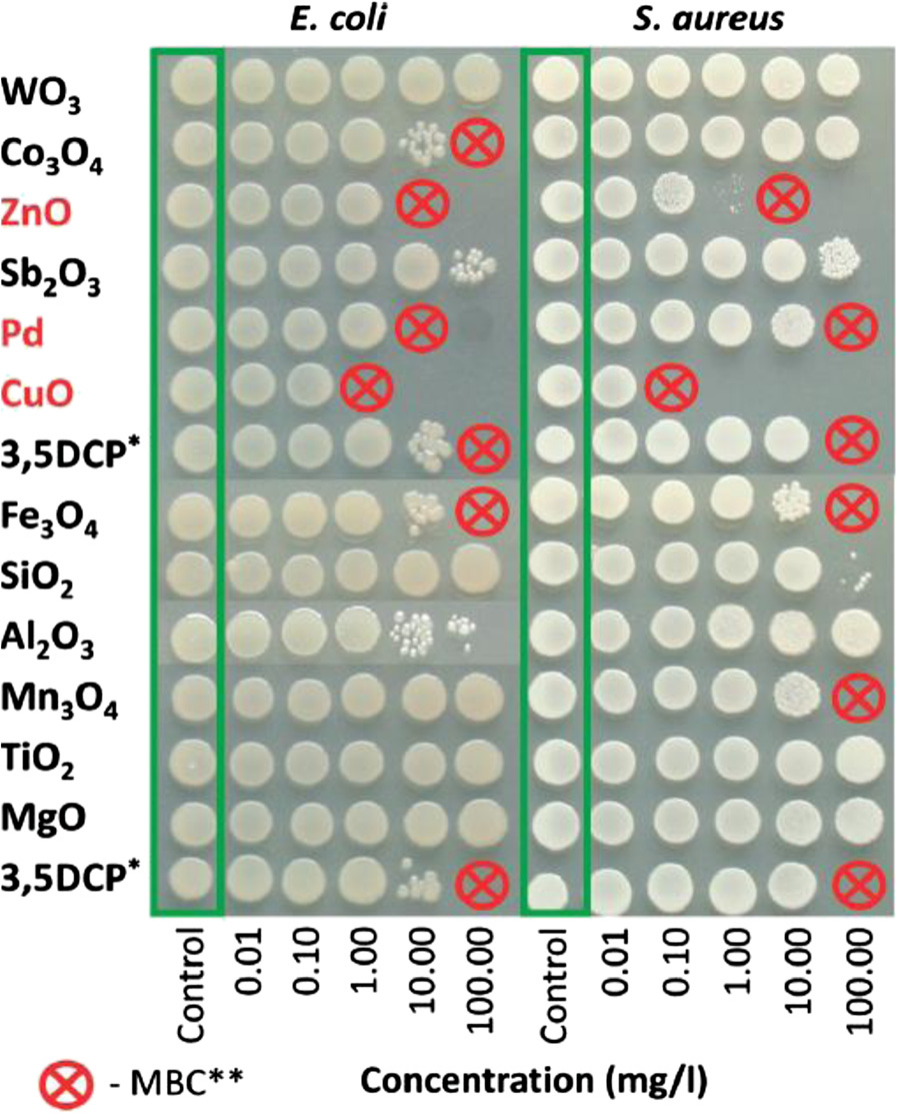
Toxicity of 12 nanoparticles to bacteria *Escherichia coli* and *Staphylococcus aureus*. Toxicity was evaluated by determining the colony-forming ability of the bacteria after exposure to nanoparticles in deionized water for 24 h at 25 °C. After exposure, 5 μl of bacterial suspension was transferred onto toxicant-free agarized LB growth medium. The concentrations of the NPs are in mg compound l^−1^. All concentrations are nominal. *3,5 Dichlorophenol was used as a positive control, **Minimal biocidal concentration [65]

Certain metal containing nanoparticles (Ag, CuO, ZnO) release metal ions and cause toxicity to bacterial cells [64, 66, 67]. Smaller nanoparticles, however, have been shown to exhibit greater toxicity, perhaps due to their penetration into the bacterial cells [68]. Of all the nanoparticles tested for toxicity, CuO was found to be most effective (Fig. 6) against *S. aureus* and *E. coli*. The other metal oxide nanoparticles inhibited the growth of these bacteria only at 100 mg L^−1^ level (Table 3). ZnO and CuO are toxic to *T. thermophila* at 6 mg L^−1^ while all other nanoparticles are toxic above 100 mg L^−1^ level which may not be found in the natural environment except in mining areas only. Since protozoa are small particle feeding organisms, they can be used to remove unwanted particles from waste water. *T. thermophila* feed on bacteria and metal oxide nanoparticles without making any distinction between the two. They get accumulated in the vacuoles of protozoa [69]. Single-wall carbon nanotubes at a concentration between 3.6 and 6.8 mg L^−1^ are ingested by *T. thermophila* after their exposure for 24 h. However, these nanoparticles are toxic above 100 mg L^−1^. *P. subcapitata* algal growth inhibition occurs by ZnO and CuO at very low level (0.1 and 0.43 mg L^−1^). The MgO and SiO_2_ are least toxic possibly because they are already present in sea water and the algae are accustomed to their presence in level below 100 mg L^−1^. Toxicity to algae is mainly due to its cells entrapped/enveloped by metal oxide nanoparticles and ROS generation [70].

**Table 3:** Categorization of nanoparticles based on the toxicity values (EC50 or MBC, mg compound l^−1^) to bacteria, protozoa and algae. All nanoparticles were tested in nominal concentrations from 0.01 up to 100 mg l^−1^ [65]

EC_50_ or MBC, mg compound l^−1^	72 h EC_50_	24 h EC_50_	30 min EC_50_	24 h MBC	24 h MBC
Organisms	Algae	Protozoa	Bacteria	Bacteria	Bacteria
Species	*Pseudokirchneriella subcapitata*	*Tetrahymena thermophila*	*Vibrio fischeri* (G^−^)	*Escherichia coli* (G^−^)	*Staphylococcus aureus* (G^+^)
Exposure Medium	Mineral Medium	DI Water	2 % NaCl	DI Water	DI Water
0.1–1	CuO, ZnO, Pd	None	None	CuO	CuO
>1–10	Co_3_O_4_, Fe_3_O_4_, Mn_3_O_4_, TiO_2_	CuO, ZnO	CuO	ZnO, Pd	ZnO
>10–100	Al_2_O_3_, SiO_2_, _WO3_	Fe_3_O_4_, TiO_2_	ZnO, Pd, WO_3_, Sb_2_O_3_	Co_3_O_4_, Fe_3_O_4_	Fe_3_O_4_, Mn_3_O_4_, Pd
>100	MgO, Sb_2_O_3_	Al_2_O_3_, Co_3_O_4_, MgO, Mn_3_O_4_, Pd, Sb_2_O_3_, SiO_2_, WO_3_	Al_2_O_3_, Co_3_O_4_, Fe_3_O_4_, MgO, Mn_3_O_4_, SiO_2_, TiO_2_	Al_2_O_3_, MgO, Mn_3_O_4_, Sb_2_O_3_, SiO_2_, TiO_2_, WO_3_	Al_2_O_3_, Co_3_O_4_, MgO, Sb_2_O_3_, SiO_2_, TiO_2_, WO

*EC*_*50*_ half effective concentration, *MBC* minimal biocidal concentration, i.e., the lowest tested nominal concentration of nanoparticles which completely inhibited the formation of visible colonies after sub-culturing on toxicant-free agarised growth medium. Prior sub-culturing bacteria were incubated with nanoparticles for 24 h at 25 °C in deionized water

The pH of the suspension containing ZnO and the algae does decrease from 8 to 4, but virtually there is no variation in toxicity as a function of pH [71]. Hartmann et al. [72] have studied the toxicity of TiO_2_ nanoparticles of 10, 30 and 300 nm against *Pseudokirchneriella subcapitata* alga. All the three types of particles exhibited algal growth inhibition. The ecotoxicity of Cd to alga, *P. subcapitata*, in presence of 2 mg L^−1^ of TiO_2_ was reduced probably due to non availability of Cd in presence of TiO_2_ nanoparticles. The toxicity was also found to be dependent on the nanoparticles and their concentration.

Ji and co-workers [73] have studied the toxicity of Al_2_O_3_, SiO_2_, ZnO and TiO_2_ nanoparticles towards green alga, *Chlorella* sp. Al_2_O_3_, SiO_2_ and TiO_2_ (DJ3, rutile) did not show significant toxicity although ZnO and TiO_2_ (HR3, anatase) inhibited the algal growth in 20 and 30 mg L^−1^ nanoparticles in aqueous solution.

The ecotoxic effects of oxide nanoparticles are dependent on their size and type. Even at very high concentration (1000 mg L^−1^), the algal growth did not show any variation from the second day to the sixth day of exposure. Nano Al_2_O_3_ showed growth promotion at the fourth day by about 19 %. Lin and Xing [74] have found nano Al_2_O_3_ as nontoxic to five plant species. However, at higher concentration of 2000 mg L^−1^ of Al_2_O_3_, root growth is inhibited [75]. Such experimental results may not be applied in the field because such a high concentration is seldom achieved in aquatic system as the algae etc. will dry up due to large accumulation of nanoparticles and other toxic materials.

The toxic effect of the nanoparticle and their bulk material are not the same. For instance, the *Chlorella* sp. toxicities for different form of Zn follow the order: Zn^2+^ > nano ZnO > bulk ZnO even when their concentrations are below 50 mg L^−1^. At higher concentration (>50 mg L^−1^), the toxicity of ZnO nanoparticles has been shown to be higher than Zn^2+^. The toxicity also depends on particle size, crystal structure, rutile and anatase. Anatase TiO_2_ is more toxic than rutile TiO_2_. Ji et al. [73] have suggested that anatase TiO_2_ release larger quantity of ROS than rutile TiO_2_ resulting in an increase toxicity [76]. However, the toxicity of anatase TiO_2_ nanoparticles decreases if their size increases above 33 nm [77]. Nano TiO_2_ and nano ZnO can produce photocatalytic ROS in presence of UV light [78], but experimental evidences demonstrated larger production of ROS even in the dark [79]. It is therefore concluded that there are other possible reasons for the toxicity of nanoparticles besides the ROS production.

## Conclusions

Algae are considered as significant nanofactories and hold a huge potential as ecofriendly and cost-effective tools, avoiding toxic, harsh chemicals and the high energy demand required for physiochemical fabrication. In the present review, we have discussed the biosynthesis of metal and metal oxide nanoparticles from a variety of algae and their toxicity against several pathogenic gram-positive and gram-negative bacterial strains. The proteins, polysaccharides, amines, amino acids, alcohols, pigments, carboxylic acids carbohydrates and sugars have been shown to act as reducing agents. Also, they act as capping and stabilizing agents for the fabricated nanoparticles. The results suggest that the functionalized metal nanoparticles may be exploited in the treatment of infectious diseases caused by bacteria and fungi. They can also be used in phytomining and sequestering metals from waste disposals by redox process.
